# Prevalence of multidrug-resistant tuberculosis among newly diagnosed cases of sputum-positive pulmonary tuberculosis

**Published:** 2011-03

**Authors:** Surendra K. Sharma, Gaurav Kaushik, Brajesh Jha, Ninoo George, S.K. Arora, Deepak Gupta, Urvashi Singh, Mahmud Hanif, R.P. Vashisht

**Affiliations:** *Department of Medicine, All India Institute of Medical Sciences, New Delhi, India*; * *Department of Microbiology, All India Institute of Medical Sciences, New Delhi, India*; ** *Department of Microbiology, Sanjay Gandhi Memorial Hospital, New Delhi, India*; † *New Delhi Tuberculosis Centre, New Delhi, India*

**Keywords:** Drug resistance, India, multidrug-resistant tuberculosis (MDR-TB), new case-pulmonary tuberculosis

## Abstract

**Background & objectives:**

The prevalence of multidrug-resistant tuberculosis (MDR-TB) is increasing throughout the world. Although previous treatment for TB is the most important risk factor for development of MDR-TB, treatment-naïve patients are also at risk due to either spontaneous mutations or transmission of drug-resistant strains. We sought to ascertain the prevalence of MDR-TB among new cases of sputum-positive pulmonary TB.

**Methods:**

This was a prospective, observational study involving newly diagnosed cases of sputum-positive pulmonary tuberculosis diagnosed between 2008 and 2009 carried out in New Delhi, India. All sputum-positive TB cases were subjected to mycobacterial culture and first-line drug-susceptibility testing (DST). MDR-TB was defined as TB caused by bacilli showing resistance to at least isoniazid and rifampicin.

**Results:**

A total of 218 cases of sputum-positive pulmonary tuberculosis were enrolled between 2008 and 2009. Of these, 41 cases had negative mycobacterial cultures and DST was carried out in 177 cases. The mean age of the patients was 27.8 ± 10.2 yr; 59 patients (27%) were female. All patients tested negative for HIV infection. Out of 177 cases, two cases of MDR-TB were detected. Thus, the prevalence of MDR-TB among newly diagnosed pulmonary tuberculosis patients was 1.1 per cent.

**Interpretation & conclusions:**

MDR-TB prevalence is low among new cases of sputum-positive pulmonary TB treated at primary care level in Delhi. Nation-wide and State-wide representative data on prevalence of MDR-TB are lacking. Efforts should be directed towards continued surveillance for MDR-TB among newly diagnosed TB cases.

The emergence and spread of multi-drug resistant tuberculosis (MDR-TB) is threatening to destabilize global tuberculosis control. The prevalence of MDR-TB is increasing throughout the world both among new tuberculosis cases as well as among previously-treated ones^1^. Although previous treatment for TB is the strongest risk factor for development of MDR-TB^2^–^4^, treatment-naïve patients are also at risk due to either spontaneous mutations or transmission of resistant strains^5^,^6^. The risk of transmission of resistant strains from close contacts is increasing day-by-day because of the growing burden of MDR-TB patients^1^. Therefore, in the present scenario, there is high likelihood that what initially seems to be drug-sensitive TB in a treatment-naïve patient might in fact be MDR-TB from the outset. Therefore, we sought to determine the prevalence of MDR-TB among new cases of sputum-positive pulmonary TB.

## Material & Methods

This was a prospective, observational study involving newly diagnosed cases of sputum-positive pulmonary tuberculosis. These are preliminary results from an ongoing double-blind, placebo-controlled trial. The cases were recruited through a dedicated chest clinic functioning at primary care level at Sanjay Gandhi Memorial Hospital in Mongolpuri, New Delhi. All suspected cases of TB attending the clinic were subjected to sputum smear examination and mycobacterial culture and drug-susceptibility testing (DST) at the New Delhi Tuberculosis (NDTB) Centre laboratory, New Delhi. The NDTB centre was accredited as the intermediate reference laboratory (IRL) during the study period. Sputum-positive pulmonary TB was defined as TB in a patient with at least 2 initial sputum smear examinations positive for acid-fast bacilli (AFB) or one sputum smear examination positive for AFB and radiographic abnormalities consistent with active pulmonary TB or one sputum smear specimen positive for AFB and culture positive for *Mycobacterium tuberculosis*^7^. New case was defined as a TB patient who has never had treatment for tuberculosis or has taken anti-tuberculosis drugs for less than one month^7^. Cultures were done on Lowenstein-Jensen (L-J) slopes by modified Petroff’s method^8^. All the isolates were identified as *M. tuberculosis* by their slow growth rate, colony morphology, inability to grow on L-J media containing p-nitrobenzoic acid (PNB), niacin test and catalase test. DST was carried out by the economic variant of 1 per cent proportion method for all drugs except pyrazinamide which was tested by the resistance-ratio method. The tested drugs and their critical concentrations (in μg/ml) were as follows: isoniazid (H)- 0.2, rifampicin (R)- 40, pyrazinamide (Z) - 100, ethambutol (E) - 2 and streptomycin (S) - 4. MDR-TB was defined as TB caused by bacilli showing resistance to at least isoniazid and rifampicin. Human immunodeficiency virus (HIV) testing was carried out routinely in all patients and HIV positive patients were excluded from the study. Written informed consent was obtained from all patients. The Ethical Committee of AIIMS hospital, New delhi approved the study protocol.

## Results

We prospectively enrolled 218 cases of newly diagnosed sputum-positive pulmonary tuberculosis between February 2008 and December 2009. Of the 218 cases, 41 patients had negative mycobacterial cultures and hence DST was carried out in 177 cases. The mean age of the patients was 27.8 ± 10.2 yr; 59 (27%) were female. The mean body mass index (BMI) was 17.33 ± 1.99 kg/m^2^. Out of 177 cases, two cases of MDR-TB were detected. Both were male, HIV negative, aged 20 and 25 yr with BMI 17.1 and 19.7 kg/m ^2^, and resistance pattern was H, R, S and H, R, E, S, respectively. Thus, the prevalence of MDR-TB among new sputum positive pulmonary TB patients was 1.1 per cent. The resistance rates (%) observed to various first-line drugs were isoniazid 6.2, rifampicin 1.1, pyrazinamide 0, ethambutol 3.4, and streptomycin 2.3. The rates of mono- and poly-drug resistance rates are shown in Table I.

**Table I:** Mono- and poly-drug resistance rates in patients

Drug	Resistance rate (%)

Isoniazid	1.7
Rifampicin	0
Pyrazinamide	0
Ethambutol	0
Streptomycin	0
Isoniazid + Ethambutol	2.3
Isoniazid + Streptomycin	0.6
Isoniazid + Rifampicin + Streptomycin	0.6
Isoniazid + Ethambutol + Streptomycin	0.6
Isoniazid + Rifampicin Ethambutol + Streptomycin	0.6

*Note:*The drug combinations not mentioned in this Table had 0%resistance.

## Discussion

We found a low prevalence of MDR-TB among new cases of pulmonary TB in Delhi. The reported prevalence of MDR-TB among new TB cases has varied from 0.14 to 5.3 per cent in previous studies from different parts of India^8^–^19^ and our findings are in consonance with such observations (Table II). But there are a few studies which have reported a high prevalence of MDR-TB among new TB cases^20^,^21^. Bias in patient selection and differences in methodology may account for such high prevalence of MDR-TB noted in these studies.

Our findings carry some important implications. Firstly, the prevalence of MDR-TB has not risen over the years, which reflects the success of DOTS in effective treatment of drug-susceptible TB and preventing the emergence of MDR-TB. Secondly, since MDR-TB is rare among new TB cases, all new cases of pulmonary tuberculosis can be treated with empirical category I regimen without the risk of treatment failures or aggravation of drug-resistance.

The major limitation of the present study is the small sample size and therefore, it is not representative of the population at large. In fact, this limitation was observed in most previous studies on MDR-TB. Nation-wide and State-wide representative data on the prevalence of MDR-TB are an urgent need of the hour to design effective empirical regimens, to monitor functioning and progress of the national TB control programme and for continued surveillance of MDR-TB among category I TB patients. In conclusion, our findings are quite reassuring in that MDR-TB prevalence has not risen over the years and still continues to be low among new cases of pulmonary TB.

**Table II:** Prevalence of MDR-TB among new cases of pulmonary TB in India reported in previous studies

Location	Period of study	No. of isolates	MDR-TB (%)

Bangalore^8^	1980s	436	1.1
Wardha^9^	1982-1989	323	5.3
North Arcot^10^	1985-1989	2779	1.6
Pondicherry^10^	1985-1991	1841	0.8
Kolar^11^	1987-1989	292	3.4
Jaipur^12^	1989-1991	1009	0.9
New Delhi^13^	1990-1991	324	0.6
Pune^14^	1992-1993	473	1.0
Tamil Nadu^15^	1997	384	3.4
North Arcot^16^	1999	282	2.8
Lucknow^20^	2000-2002	318	13.2
Hyderabad^17^	2001-2003	714	0.14
Ernakulam^18^	2004	305	2.0
Gujarat^19^	-	1571	2.4
Mumbai^21^	2004-2007	493	24
Present study	2008-2009	177	1.1
